# The pathogenicity and virulence of *Toxoplasma gondii*

**DOI:** 10.1080/21505594.2021.2012346

**Published:** 2021-12-11

**Authors:** Syrian G. Sanchez, Sébastien Besteiro

**Affiliations:** Lphi, UMR5235, Univ Montpellier, CNRS, Montpellier, France

**Keywords:** *Toxoplasma gondii*, chronic toxoplasmosis, acute toxoplasmosis, parasite transmission, secreted virulence factors, immune evasion

## Abstract

*Toxoplasma gondii* is a parasitic protist infecting a wide group of warm-blooded animals, ranging from birds to humans. While this infection is usually asymptomatic in healthy individuals, it can also lead to severe ocular or neurological outcomes in immunocompromised individuals or in developing fetuses. This obligate intracellular parasite has the ability to infect a considerable range of nucleated cells and can propagate in the intermediate host. Yet, under the pressure of the immune system it transforms into an encysted persistent form residing primarily in the brain and muscle tissues. Encysted parasites, which are resistant to current medication, may reactivate and give rise to an acute infection. The clinical outcome of toxoplasmosis depends on a complex balance between the host immune response and parasite virulence factors. Susceptibility to the disease is thus determined by both parasite strains and host species. Recent advances on our understanding of host cell-parasite interactions and parasite virulence have brought new insights into the pathophysiology of *T. gondii* infection and are summarized here.

## Introduction

*Toxoplasma gondii* belongs to the phylum Apicomplexa, a diverse group of protists that are mostly intracellular parasites and can cause potentially serious disease in animals and humans. *T. gondii* is arguably the most widespread of these, being able to infect almost any warm-blooded vertebrate, and thus a common cause of infection in wild and domestic animals, in addition to an estimated one-third of the human world population [1]. Seropositivity rates in the human population are evolving, and they range from less than 10% to over 90% depending on the country or region considered, varying in part because of regional socioeconomic parameters and population habits [2]. There is, for example, a higher prevalence in South America, Central America, and continental Europe, than in the United States of America or the United Kingdom. Seroprevalence is also widespread in wild and domestic animals, which are important as reservoirs for *T. gondii*, but also as sources for human contamination through meat consumption [3]. Toxoplasmosis in farm animals is not only a problem for human contamination but it also has a considerable burden on the livestock (on milk production and reproductive performance for instance) and thus represents a significant cost for the industry [4].

While a single species has been described for the genus *Toxoplasma*, there are several clonal lineages that differ in their pathogenicity. In Europe and North America the population structure of *T. gondii* is dominated by four main clonal types (I, II, III, and XII) [5–7]. In Europe, type II strains (as well as type III, although to a lesser extent) are the most prevalent in a wild and domestic environment [8,9]. In North America, domestic isolates are similar to those in Europe (types II and III), but in the wild environment strains belonging to type XII predominate [7,10]. In other parts of the world, the situation is more contrasted. In South America, for example, there is a much greater genetic diversity [11–13], suggesting a greater occurrence of recombination. Strain virulence is typically defined by the outcome of infection in the mouse model, in which type I strains are much more virulent, than type II and III strains [14]. The type of *T. gondii* strain also has a considerable impact on the pathogenicity, with, for example, severe cases of acquired toxoplasmosis in immunocompetent patients caused by highly pathogenic South American strains from the wild [15].

Upon infection by *T. gondii*, disease development also depends on the type of host, its genetic background, and of course its immune status. Several species seem to be naturally resistant to *T. gondii* infection, while others are very susceptible, due in part to factors including the proximity of their habitat with the definitive hosts of the parasite [16]. Yet, one of the most critical factors influencing susceptibility to *T. gondii* remains the host immune system and the way it is modulated by parasite factors [17–20]. In this review, we will summarize the recent findings on the host- and parasite-dependent factors that not only govern the outcome of infection, but also give clues as to why *T. gondii* is such a highly successful parasite.

## *T. gondii* life cycle and routes of transmission

**Figure 1.**
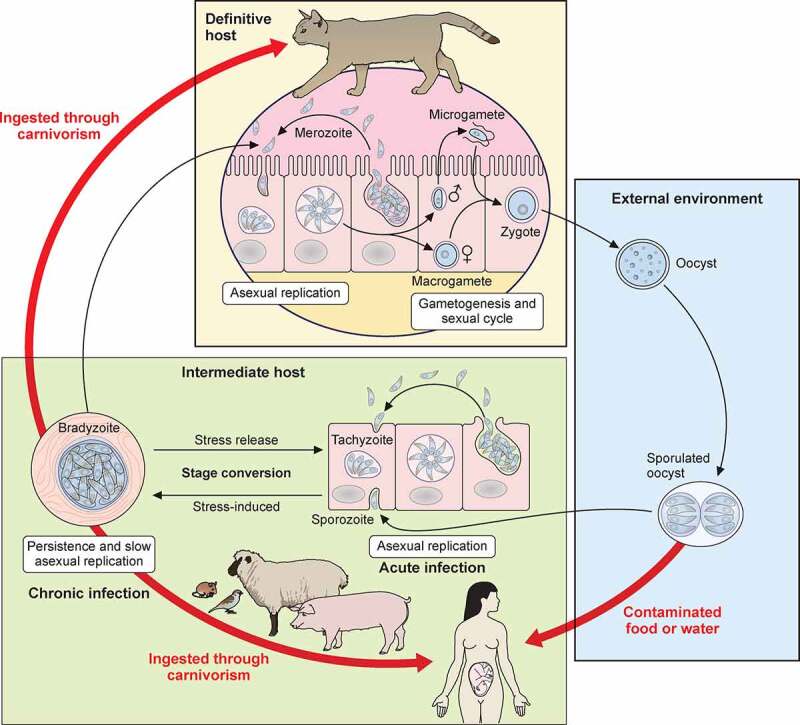
Life cycle of *T. gondii*. Schematic representation of the infective stages and their modes of transmission and replication in their respective hosts

The life cycle of *T. gondii* (Figure 1) involves both sexual replication in felids (definitive hosts), and asexual replication in a variety of vertebrate hosts (intermediate hosts). Felids ingest the parasite by preying on infected intermediate hosts that contain encysted bradyzoites. Bradyzoites are released from cysts under the action of intestinal enzymes and acid digestion, and invade the epithelial cells of the small intestine. Although the parasite may disseminate throughout the definitive host’s body and give rise to clinical signs, it is rarely the case [21]. More frequently, in the intestine and within the course of a few days, bradyzoites will develop into different morphological enteroepithelial stages (or schizonts) to finally reach the merozoite stage [22]. In turn, after a few rounds of asexual division merozoites will differentiate into male (micro-) and female (macro-) gametes. Male and female gametes will then fuse to produce diploid oocysts, which will be encapsulated in a thick impermeable wall. Millions of these will be shed in the feces of the felid and contaminate the environment. There, oocysts undergo a sporulation process involving meiosis and mitosis to generate mature and infectious haploid sporozoites within now so-called sporulated oocysts [23]. The oocysts are remarkably resistant and can persist in the environment for a long period of time, which allow their dissemination in the terrestrial or aquatic environments [24,25]. Intermediate hosts ingest sporulated oocysts through contaminated food (such as produce) or water. Sporozoites will invade host cells and occupy a transient parasitophorous vacuole (PV) in which they quickly differentiate into the tachyzoite form [26]. Tachyzoites are highly proliferative and invasive forms that will disseminate in the host, and they are responsible for the symptoms of acute toxoplasmosis. They can travel through blood vessels or the lymphatic system and reach a number of different locations, like visceral organs, muscle, and nervous tissue. The hijacking of host immune cells allows parasite dissemination through the body [27], and this way (but also using paracellular entry and transcellular migration [28,29]) they can also cross non-permissive biological barriers, like the blood–brain barrier, to reach immune-privileged organs like the brain. In fact, immunocompetent individuals will eventually control this acute phase of infection, but coincident to the emergence of the host immune response, fast-replicating tachyzoites will differentiate into slow-growing encysted forms called bradyzoites that will remain largely hidden from the immune system [30]. These persistent forms reside primarily in the central nervous system and muscle [31], where they may remain for a very long time [32]. This ensures parasite transmission to the definitive host to complete the cycle, at least when felids can prey on the intermediate host. When intermediate hosts are not typical preys of felids, the parasites can still be transmitted to other intermediate hosts by carnivorism, maintaining a parasite transmission cycle without need of sexual replication.

Human contamination can thus happen through food consumption: by ingestion of uncooked or undercooked cyst-containing meat, or of sporulated oocysts-containing vegetables, fruits or water [33]. For this reason, adequate sewage or water treatment, and washing of produce and proper cooking of meat, are important to prevent foodborne toxoplasmosis [34]. Other routes of transmission include congenital transmission of tachyzoites from a primarily infected mother to the developing fetus through the placenta [35]. Preventative measures for limiting the contact of the mother with known routes of transmission during pregnancy, as well as prompt diagnosis, and rapid initiation of medical treatment at the onset of infection, are critical for managing congenital toxoplasmosis [36]. Another potential source of contamination in humans, although rare, is from blood transfusion [37] or organ transplantation [38] from infected donors.

## Clinical manifestations of toxoplasmosis

Toxoplasmosis is an infection that is usually asymptomatic or may result in a mild, self-limiting illness in immunocompetent individuals. However, in immunocompromised individuals or fetuses it can lead to much more serious clinical manifestations [39].

Congenital toxoplasmosis can occur when there is primary maternal infection during pregnancy, as during the parasite dissemination phase it may cross the placental barrier to contaminate the developing fetus. It can cause neurological, ocular, or systemic damage with variable severity, which depends on the gestational age at the time of primary maternal infection. For instance, first-trimester maternal infection can lead to more severe manifestations [40]. The most important sequelae for the newborn include hydrocephalus, mental retardation, epilepsy, and blindness, although some of these can also occur later in life [41].

In adults, immunodeficiency can also lead to severe toxoplasmosis, which is most often the result of reactivation of latent infection, even if acute acquired infection may also occur. Individuals who are immunocompromised or immunosuppressed (in the context of HIV infection [42], or for cancer patients [43] and transplant recipients) are particularly at risk. The most serious outcome in this context is arguably toxoplasmic encephalitis, in which recurrence of toxoplasmosis from parasites encysted in the central nervous system can lead to substantial tissue damage and inflammation [44]. Common symptoms include headache, fever, ataxia, or seizure, but this cerebral form of toxoplasmosis can be potentially life-threatening if not treated.

There is also an ocular presentation of the disease called ocular toxoplasmosis, a progressive necrotizing retinitis, that may lead to vision-threatening complications [45]. This can happen both in the context of congenital or acquired toxoplasmosis. As with the brain and muscles, the eyes are one of the organs the tachyzoites can disseminate to upon initial infection. There, they can cause self-limiting lesions, but might encyst and be able to subsequently reactivate if host immunity becomes impaired. Although this is for the moment poorly understood, recurrences may also occur in immunocompetent subjects, where *T. gondii* is a major cause of posterior uveitis worldwide [46]. Of note, higher severity and frequency of ocular toxoplasmosis in South America compared with Europe is probably due to exposure to more virulent strains [47,48].

In addition to the obvious deleterious effects of acute toxoplasmosis, chronic toxoplasmosis (i.e. the long-term persistence of the parasite in the form of tissue cysts), especially as it targets the central nervous system, may also have an important impact on behavioral changes and psychiatric disorders [49]. One famous example of behavioral alteration is how the parasite presence in the brain of the intermediate hosts may decrease felid aversion, or at least induce a general boldness (including toward predators), which may facilitate the completion of the life cycle and thus favor the reproductive efficiency of the parasite. This has been observed in laboratory settings for rodents [50,51], and even in the wild for larger animals [52]. Epidemiological studies and meta-analyses have shown that *T. gondii* seropositivity can be associated with a number of mental health disorders, including schizophrenia, but also epilepsy and neurodegenerative diseases [53], although causality has not been firmly established. So far, there is no clear experimental demonstration of the direct effects of *T. gondii* on neurons and their functions, and few evidence of the cellular mechanisms that are dysregulated by the long-term establishment of this parasite in the brain [54].

## Typical ultrastructure of a *T. gondii* invasive stage

**Figure 2.**
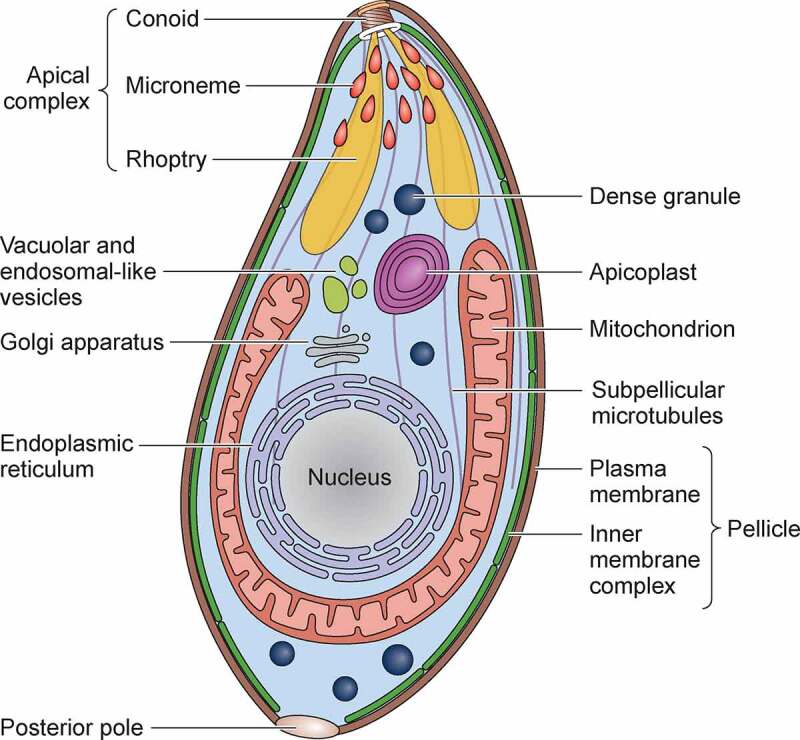
Ultrastructure of a *T. gondii* tachyzoite. As displayed on this schematic representation tachyzoites, like other *T. gondii* invasive zoite stages, are highly polarized cells and contain specialized organelles involved in the secretion of virulence factors

*T. gondii* is an obligate intracellular parasite that has the remarkable ability to invade a variety of nucleated cells. The parasite encounters multiple host cell and tissue types during its life cycle, which involves four different invasive forms: the tachyzoite, bradyzoite, sporozoite and merozoite [23]. All the infectious stages of the parasite present the same overall organization, and are highly polarized cells displaying an elongated shape (Figure 2). They contain universal eukaryotic organelles such as a nucleus, endoplasmic reticulum and Golgi apparatus, but also have some more original features. For instance, *T. gondii* tachyzoites harbor two organelles of endosymbiotic origin: a mitochondrion, essentially found as a single ramified organelle [55], although its morphology is dynamic in nature [56]; and a non-photosynthetic plastid named the apicoplast, originating from a secondary endosymbiotic event, and thus enclosed by four membranes [57]. Both organelles have important metabolic functions for the parasite [58]. Another similarity with plants is the vacuolar/lytic compartment of tachyzoites, that may also be involved in osmoregulation [59,60]. Tachyzoites are enclosed by a trilaminar membrane structure termed the pellicle, that apicomplexan parasites share with other alveolates (like ciliates and dinoflagellates) [61]. This structure comprises the plasma membrane and the underlying inner membrane complex, which is made of flattened membrane sacs. It is supported on its cytoplasmic face by a complex and highly organized network of intermediate filament‐like proteins and by a subpellicular network of microtubule cytoskeleton, which is instrumental in driving the gliding motility of the parasite [62]. The apical complex, which gave its name to the Apicomplexa phylum, comprises a cytoskeletal structure called the conoid. It is an assembly of spirally arranged fibers originating from the preconoidal rings, at the distal tip of the structure, but also the polar ring, from which the 22 subpellicular microtubules originate, and two short intraconoidal microtubule [63,64]. This structure is closely associated with two different types of specialized apical secretory organelles called micronemes [65] and rhoptries [66,67], that secrete virulence factors that are essential for dissemination and survival of the parasites. Dense granules are another type of specialized secretory organelle that will later on release important factors for the intracellular establishment of the parasite [68], although these particular vesicles are not restricted to its apical part [69].

## Host cell invasion and parasite development: The example of tachyzoites

**Figure 3.**
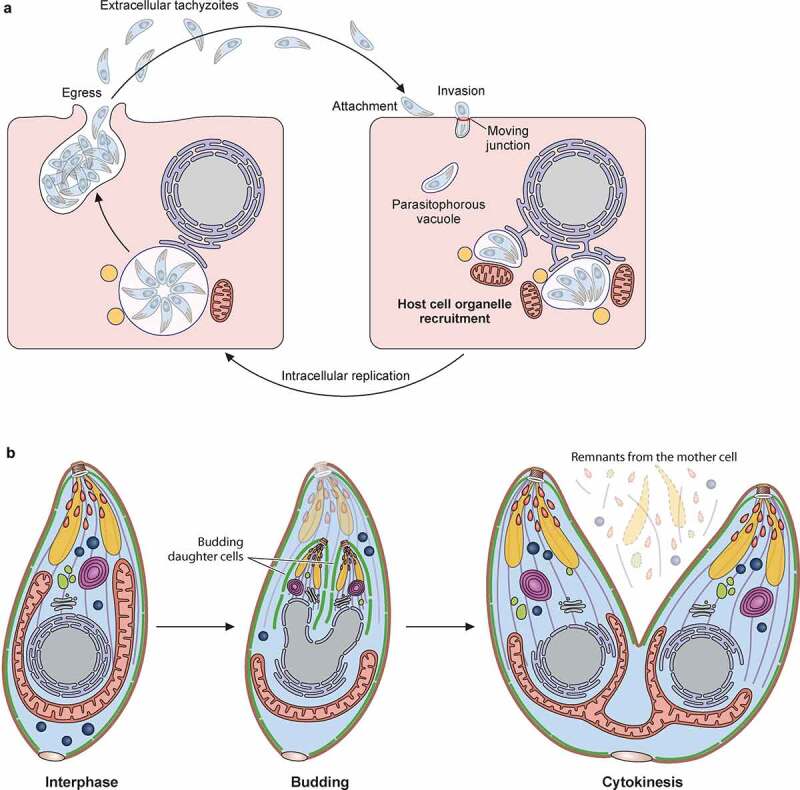
Asexual replication of *T. gondii* tachyzoites. A) Schematic representation of the lytic cycle of *T. gondii* tachyzoites, which comprises three main steps: invasion, intracellular replication and egress. B) Schematic representation of endodyogeny, the process by which *T. gondii* tachyzoites replicate intracellularly. It involves the coordinated assembly and internal budding of two daughter cells within a mother cell. The daughter-forming material is either synthesized *de novo* or recycled from the mother cell

Host cell invasion followed by rapid asexual multiplication are key steps of the *T. gondii* life cycle, which allow for instance population expansion in the definitive or intermediate hosts, through the merozoites and tachyzoites developmental stages, respectively (Figure 1). The invasion process, and subsequent steps of intracellular asexual replication and exit (or egress) from the host cell, collectively referred to as the “lytic cycle” [70], is mostly studied for tachyzoites (Figure 3a). Host cell invasion mechanism involves the formation of a PV that constitutes a unique replicative niche, providing some protection from the host cell and access to nutrient sources [71,72]. Invasion and subsequent establishment of the parasite in the PV is possible thanks to the sequential exocytosis of the three aforementioned specialized secretory organelles: micronemes, rhoptries, and dense granules [73]. Micronemes are rod-like organelles clustered at the apical pole of the parasites (Figure 2), they contain a large array of proteins (MICs), many of which are important for the invasion process. Several MICs secreted by extracellular tachyzoites are adhesins that can bind to a number of different host cell surface components that comprise proteins, but also carbohydrates [74]. This mediates an important initial attachment step for the parasites at the surface of the host cell and provides an anchor for gliding motility, which is crucial to the invasion process [75]. Some MICs seem also important for controlling the exocytosis of the rhoptries [76], which are also apical secretory organelles, and are acting downstream in the process of invasion. Rhoptries are club-shaped organelles whose protein content localizes to discrete sub-compartments: the bulbous part, containing proteins called ROPs that are involved in the subversion of host cell functions; and the neck, containing proteins called RONs that are more specifically associated with host cell invasion [67]. Rhoptries and micronemes are both involved in the secretion of factors that will constitute the moving junction (MJ), a structure formed by a MIC ligand and a RON receptor protein complex secreted by the parasite into its host cell plasma membrane to anchor itself firmly prior to entering [77]. The MJ also constitutes a physical barrier that likely restricts the incorporation of host plasma membrane proteins into the nascent PV membrane (PVM) that forms as the parasite enters the cell [78,79]. This selective incorporation of host material is critical for rendering the PV nonfusogenic with the host endolysosomal system, and thus preventing parasite degradation by lysosomal acidification [78].

Later during the invasion process, or once they are intracellular, the parasites will secrete other factors that will help modifying the host cell in order to facilitate replication [80]. These include ROPs and dense granule effectors (GRAs), which are secreted in the vacuolar space or beyond, like the PVM, the host cytosol, or even the host nucleus [81]. Some GRAs are involved in the genesis of an intravacuolar membrane network [82] as early as 10–20 minutes post-invasion, or the establishment of pores in the PV to function as molecular sieves [83]. These modifications are important to the intracellular growth of the parasites, as they will allow the scavenging of essential nutrients from the host. *T. gondii* is auxotroph for a number of important metabolites, including amino acids, nucleotide precursors, essential co-factors and lipids. Thus to survive and ensure its division it must acquire these molecules from its host [84]. One striking feature that also quickly follows invasion is the recruitment of host organelles (Figure 3a) around the parasite-containing PV. These include part of the endoplasmic reticulum, mitochondria, but also multivesicular bodies and transport vesicles, Golgi ministacks and lipid droplets [85], and GRAs are instrumental in this process. For instance, the anchorage of host mitochondria to the PVM is mediated by a GRA protein called MAF1 (for mitochondrial association factor 1) [86]. Recruitment of host organelles by the parasites may serve several purposes including the scavenging of nutrients, but also the counteracting of harmful host functions. Interestingly, like several other host-related features controlled by GRA and ROP effectors (which are described later in this review), the organelle recruitment capacity shows some difference between different strains [86], which may also account for strain-specific differences in pathogenesis.

Tachyzoites replicate asexually through a process called endodyogeny (Figure 3b), in which two daughter cells are assembled inside a mother cell that ends up being consumed as they are formed, leaving behind only a small residual body [87]. In this particular form of replication, daughter cell budding and DNA replication are coordinated, and while some organelles are synthesized *de novo* (like rhoptries and micronemes), others like the apicoplast or the Golgi apparatus are duplicated and segregated into nascent daughter buds in a process coordinated by the centrosomes [87,88]. After successive rounds of division (usually 5 or 6, at least *in vitro* [89,90]) tachyzoites will actively egress the PV and the host cell [91,92], thereby causing its destruction. Like invasion, egress is highly dependent on microneme secretion, which is regulated through calcium release [93,94], itself depending on a signaling cascade sensing local concentration of phosphatidic acid [95,96]. Among the microneme-dependent factors important for egress are those governing parasite motility [62], but also specific factors like the PLP1 perforin that destabilizes membranes and facilitates the exit of the parasites [97]. Although some laboratory-adapted strains show some short-term survival (a few hours) in the extracellular environment, once they have egressed, extracellular tachyzoites must invade a new host cell in order to survive and initiate a new lytic cycle (Figure 3a).

## Replication of other developmental stages

As tachyzoites can be easily propagated *in vitro* their division process is well characterized, but the multiplication of other parasite stages is often less studied, although they are also critical for the pathogenicity of *T. gondii* (Figure 1). The bradyzoites, the latent and persistent stage found in the intermediate host, also replicates asexually, albeit much more slowly and asynchronously than the tachyzoite stage [98]. It does so by a combination of endodyogeny (like for tachyzoites) and also, occasionally, by a process called endopolygeny, by which more than two daughter cells form within the mother cell [99]. The asexual expansion in enterocytes of the small intestine of the definitive host is also an important prerequisite to the formation of gametes and for the hundreds of millions of oocysts that will be shed in the environment subsequently. It involves a complex differentiation through five morphologically-different stages, some of which divide by endodyogeny, whereas others multiply by endopolygeny, or schizogony (with multinucleated intermediates, or schizonts). At the end of this division process, the daughter parasites will bud from the periphery to produce infective merozoites [22]. After this asexual multiplication, the sexual cycle starts by gametogenesis and the formation of macro- and microgamonts, that will later develop into gametes. Macro- and microgametes are not equilibrated in numbers, and the rate of macrogamete fertilization is unknown. However, *T. gondii* has clearly managed to achieve maximum oocyst output, with hundreds of millions of them potentially being shed by a single felid [100]. Many questions still remain regarding these understudied stages [100,101]. Yet, the recent discovery that linoleic acid is critical in conferring the specificity of felids as definitive hosts has allowed using mice as efficient oocysts spreaders when their diet is supplemented with an excess of this fatty acid [102]. This new experimental model will likely help solving some of the existing conundrums regarding the part of the *T. gondii* life cycle that takes place in the definitive hosts.

The conversion between the different developmental stages is also key for the pathogenicity, and our understanding of gene regulation across these life stages has recently advanced. For example, recent studies have identified major transcriptional regulators controlling sexual commitment [103] or encystation [104], providing access to new tools for characterizing stage-specific transcriptomes. Transcriptomic and proteomic analyses have also highlighted that different *T. gondii* infectious zoite stages express specific repertoires of genes [105–109]. Noticeably stage-specific effectors include different subsets of GRAs (some of which are for example involved in the formation of the cyst wall that surrounds bradyzoites [110]), MICs and ROPs, which are important in attachment, invasion, and host cell modification. This is perhaps unsurprising, as it may reflect the capacity for the different zoite stages to invade and develop into different host cell types. From this point of view, tachyzoites are clearly the most versatile, and yet these stages also show some strain-specific differences in the virulence factors they express. Some of these virulence factors inhibit host defense mechanisms and thus contribute to the relative differences in virulence during primary infection.

## Immune response against *T. gondii* in the intermediate host

The ability of *T. gondii* to persist in a wide range of intermediate hosts is the result of a balance between the host immune system and the parasite’s own escape mechanisms. Noticeably, cell responses to infection are dependent on species and cell types infected by the parasite. It is also known that the different parasite strains will not induce the same immune response depending on the presence and the polymorphism of their effectors. Most of the *in vivo* infection data for *T. gondii* were generated in mice, not only because they are well-characterized models for mammalian immune function in general, but also because they are natural hosts of the parasite. Yet, although the findings described in this part mostly focus on mice as the archetypal model for mammalian response to *T. gondii*, it should be kept in mind that there are marked differences between humans and mice in sensor and effector proteins that determine host resistance to this parasite.

### Initial recognition of T. gondii

**Figure 4.**
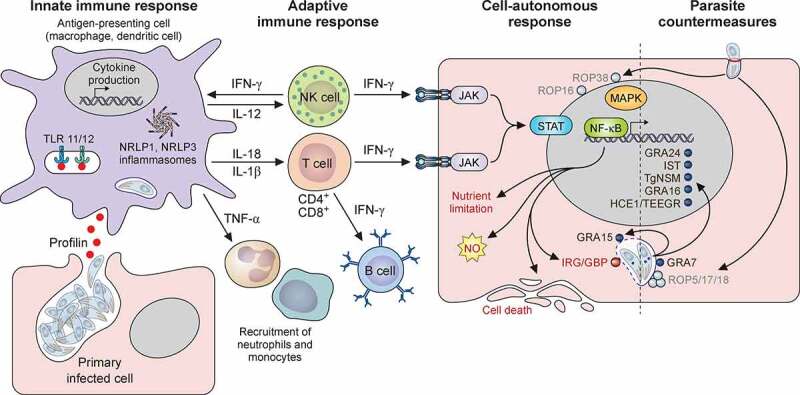
Host immune response to *T. gondii* infection and examples of parasite evasion mechanisms. Schematic view of typical components of the murine immune response to *T. gondii* upon initial infection. Cells involved in the innate and adaptive immune response, through the secretion of pro-inflammatory cytokines like IL-12, will elicit an IFN-γ-dependent activation of various cell-autonomous pathways for limiting parasite growth, which include parasitophorous vacuole destruction by immunity-related GTPases, nitric oxide production, nutrient limitation and host cell death. Virulent parasite strains can in turn secrete factors from their rhoptries or dense granules, that will interfere with nucleus-located upstream transcriptional regulators of the immune response, or with parasitophorous vacuole-located host effectors

In the early stages of *T. gondii* infection, dendritic cells (DCs), macrophages, and monocytes are the first host cells to respond (Figure 4). Classically, during pathogen infection the host will first identify the “non-self” via receptors called PRRs (pattern recognition receptors) located on the cell surface or inside the cell (like Toll-like receptors – TLR-). These receptors will generally recognize components of microbes or pathogens called MAMPs or PAMPs (microbes/pathogens associated molecular patterns). This way, innate immune cells will trigger the production of IL-12, a cytokine that plays an early and major role in the resistance to bacterial and parasitic infections [111]. In mice, the main mechanism driving IL-12 production in response to *T. gondii* infection is through the recognition of *T. gondii* profilin by TLR11 and TLR12 [112–114]. However, other proteins or parasite molecules, such as glycosylphosphatidylinositols, can also activate TLRs [115–117]. Noticeably, mice deficient for myeloid differentiation primary response 88 (MyD88), an important adaptor for signaling by most TLRs, are highly susceptible to *T. gondii* infection [118]. Humans do not have functional equivalents to all murine TLRs, and thus may not use the exact same mechanism for parasite sensing [119], which might involve sensing of parasite-derived RNA and DNA instead of profilin [120]. Yet, importantly, IL-12 is also produced by human innate immune cells in response to *T. gondii* [121].

IL-12-producing cells such as DCs and macrophages are important actors for controlling the parasite at the early onset of infection, but various other cell types, including neutrophils and inflammatory monocytes, are also involved (Figure 4). This also depends on the parasite tropism and site of infection. For instance, when intermediate hosts are infected through the ingestion of contaminated food, primary infected cells include intestinal epithelial cells and peritoneal cells. Innate lymphoid cells (ILCs) produce high levels of interferon-gamma (IFN-γ) and tumor necrosis factor, and help protecting against *T. gondii* infections in the intestine and in secondary lymphoid organs [122,123].

Other components of the innate immune system that can limit parasite growth are multiprotein complexes called the inflammasomes. They include sensor proteins that can detect a number of environmental and microbial danger signals, and will then in turn activate caspase-1, a protease that will allow cleavage and release of proinflammatory cytokines such as IL-1β and IL-18, but also induce host cell death by a specific process called pyroptosis [124,125]. NLRP1 (nucleotide-binding domain leucine-rich repeat pyrin domain containing 1) and NLRP3 are PRRs that were found to be important *in vivo* regulators of *T. gondii* proliferation [126,127]. NLRPs and the inflammasome cascades they induce have essentially been described in DCs and macrophages, but they are also present in other cell types including intestinal epithelial cells or polynuclear neutrophils. As *T. gondii* can potentially infect many nucleated cell types, the inflammasome is thus likely important for limiting parasite growth and dissemination in several tissues.

### The adaptive response to infection

Pathogen-activated antigen-presenting cells, particularly macrophages and DCs will induce the proliferation and stimulation of natural killer (NK) cells through IL-12 production in conjunction with TNF-α [128], and this is enhanced by IL-18 production [129] (Figure 4). This will trigger a typical T helper type 1 (Th1) effector response, with IFN-γ-producing CD4 + T cells, and cytotoxic CD8 + T cells. IFN-γ is clearly the main mediator for resistance to *T. gondii* [130]. A robust IFN-γ-dependent adaptive Th1-immune response is likely important in both mouse and humans for controlling parasite proliferation [130,131]. Moreover, it is not only crucial for the resolution of acute infections, but also for the control of the latent chronic infections [132].

It should be noted that besides the paramount importance of cellular immunity, in the context of murine toxoplasmosis, humoral immunity, and antibodies-generating B cells have also been shown to contribute at least in part to the control of long-term parasite persistence and vaccination-induced resistance to the infection [133–135].

### The cell-autonomous immune response against T. gondii

Cytokines help mounting an efficient anti-parasitic response by activating cells through specific receptors. One of the best-characterized pathway is the Janus kinase/signal transducers and activators of transcription (JAK/STAT) signaling pathway, which can lead to the transcriptional modulation of hundreds of IFN-regulated genes [136,137]. STAT1 and STAT4, for instance, influence the transcription of pro- but also anti-inflammatory molecules, and are important for innate NK and adaptive T cell responses involved in the resistance to *T. gondii* [138,139]. Yet, beyond the well-known and specialized set of immune cells, most cell lineages are able to defend themselves against infection through a number of processes collectively termed cell-autonomous immunity [140] (Figure 4), for which IFN stimulation plays a considerable role [141]. One of the best characterized IFN-γ-stimulated process of *T. gondii* elimination in mice is through the action of two types of GTPases: Immunity Regulated GTPases (IRGs) and Guanylate Binding Proteins (GBPs). These proteins are recruited to the PVM through a complex and sequential process involving several autophagy-related proteins (acting through an atypical non-degradative function [142]), as well as ubiquitin and p62 [143]. The GTPases seem to disrupt the vacuolar membrane by a process that is still not completely characterized, subsequently exposing the parasites for degradation [144]. Human cells have a wide repertoire of GBPs, but they do not express the IRGs usually found in mice [145], so there is a difference between these two hosts in the GTPase effectors involved in the control of parasite growth. There is generally less information available on the human cell-autonomous response against *T. gondii*. For example how the IFN-γ-stimulated non-canonical autophagy pathway mediates parasite growth restriction in HeLa cells is not completely elucidated [146,147].

Cell-autonomous defense mechanisms also include nutrient limitation and other anti-parasitic strategies [141], although they have been mostly characterized in macrophages. For instance, in IFN-γ-stimulated murine macrophages, nitric oxide (NO) limits the replication of *T. gondii* [148], while other reactive oxygen species can be used for the clearance of some of the less virulent parasite strains, even by non-stimulated macrophages [149]. IFN-dependent stimulation of indoleamine 2,3‑dioxygenases (IDOs) leads to tryptophan depletion (an amino acid the parasite is auxotrophic for) and restricts parasite growth; this has been characterized in human macrophages [150], but also fibroblasts [151]. Yet again, IDOs do not seem to have such a strong implication in *T. gondii* clearance in the mouse model [152,153], highlighting possible differences between hosts in parasite control strategies. As another striking example of this, limitation of iron supply, but not tryptophan, seems to be the main nutrient deprivation restricting parasite growth in IFN-γ-stimulated primary rat enterocytes [154]. It should thus be noted that many parasite-restricting processes have been studied in only a few mammalian hosts and certain cell-types, so to which extent they can be extrapolated to other cell types remains uncertain.

## Parasite countermeasures to the host immune response

As we have seen before, *T. gondii* contains several secretory organelles that release virulence factors crucial for host cell invasion and establishment of the parasite in a PV. As early as the invasion process begins, one of the first protective measure is the formation of the MJ that, as a molecular sieve, ensures that the nascent PV will be nonfusogenic with host lysosomes and endosome [78], and thus maintains a neutral pH [155]. Then, the rhoptries and later the dense granules, subsequently help the *T. gondii* tachyzoites modulating the host immune response and defense mechanisms by secreting GRA and ROP effectors, respectively, beyond the PVM (selected factors are highlighted on Figure 4, but more details can be found in recent reviews on this topic [17–20]). Because of the broad host range of *T. gondii*, it must be equipped to intersect with a wide variety of evolutionarily distinct immune systems and host cell types. Again, it should be noted that most of the studies on parasite modulation of the host immune response were performed in the mouse model, and likely involve a different set of effectors depending on the host and the cell type. Moreover, many of these factors, which are instrumental in defining the virulence profile of the parasites for mice, act in a strain-specific manner (Table 1).

**Table 1.** Secreted *T. gondii* ROP and GRA effectors and their strain-specific impact on the host. Abbreviations for cellular localizations: ER, endoplasmic reticulum; IVN, intravacuolar membrane network; PV, parasitophorous vacuole; PVM, PV membrane; Acronyms for host target proteins: ASC, Apoptosis-associated Speck-like protein containing a CARD (caspase activation and recruitment domain); ATF, activating transcription factor; CAMLG, calcium-modulating ligand; CCCL, C-C motif chemokine ligand; CXCL, C-X-C motif chemokine ligand, DP1, E2F dimerization partner 1; E2F, E2 transcription factor; GBP, guanylate-binding protein; GSK3, glycogene synthase kinase 3; HAUSP, herpesvirus-associated ubiquitin-specific protease; IRG, immunity-related GTPase; MAPK, mitogen-activated protein kinase; MIB complex, mitochondrial intermembrane space bridging complex; NCOR1, nuclear receptor corepressor 1; NuRD, nucleosome-remodeling deacetylase; PP2A, protein phosphatase 2A; STAT, signal transducers, and activators of transcription; TRAF; TNF receptor associated factor

Effector name	ToxodB Accession Number (Type I)	Localization	Target host protein	Role	Type I	Type II	Type III	Ref
**ROP effectors**								
ROP5	TGGT1_308090	PVM	Irga6	Inhibition of PVM IRG coating and activation of ROP18	Active	Less active (ROP18 activity not enhanced)	Active	[156–159]
ROP16	TGGT1_262730	Nucleus	STAT3/6	Activation of STAT3/6 inducing a decrease of IL-12 expression and suppressing TH1 response. Reduces the PVM coating of GBPs	Active	Less active (no sustained STAT3/6 activation)	Active	[160,181,204]
ROP17	TGGT1_258580	PVM	Irga6/Irgb6	Enhances ROP18 activity and binds IRGs (preferentially Irgb6) for disassembly	Active	Not studied	Not studied	[184]
ROP18	TGGT1_205250	PVM/ER	IRGs, ATF6β	Binds IRGs for disassembly (preferentially Irga6). Also targets host transcription factor ATF6β, reducing antigen presentation	Active	Active	Less active (low expression)	[160–164,200–202]
ROP38	TGGT1_242110	IVN/PVM	Unknown	Inhibits MAPK/NF-κB pathways, controlling apoptosis in infected cells and IL-18 secretion. Expression levels vary between *T. gondii* strains	Potentially less active (low expression)	Active	Active (high expression)	[165–167,183]
ROP54	TGGT1_210370	PVM	GBP2	Inhibits GBP2 coating at the PVM	Less active? (no virulence phenotype for the KO)	Active	Not studied	[168]
**GRA effectors**								
GRA6	TGGT1_275440	IVN/PVM	CAMLG	Activates host transcription factor NFAT4 via CAMLG, leading to the expression of CCCL2/CXCL2 and neutrophil/monocyte recruitment. In type II parasites, has an epitope eliciting T-cell response	Active	Less active but has an epitope inducing a strong T-Cell response	Active	[169,170]
GRA7	TGGT1_203310	PVM	Irga6, TRAF6, ASC	Accelerates the turnover of Irga6 by interacting with the ROP5/ROP18 complex. The GRA7 protein can also stimulate the immune system though TRAF6/ NF-κB activation and inflammasome activation through the ASC adaptator.	Active	NF-κB pathway and macrophage activation	Not studied	[164,171,197,199]
GRA12	TGGT1_288650	IVN/PVM	Unknown	Inhibits IFN-γ mediated parasite killing	Active	Active	Not studied	[172]
GRA14	TGGT1_239740	PV/PVM/IVN	Unknown	Activation of NF-κB pathway and recruitment of macrophages in type II parasites, potentially in other strains too	Active	Active	Not studied	[173,199]
GRA15	TGGT1_275470	PVM	TRAF2/TRAF6 GBP1	In type II parasites, activation of NF-κB pathway via TRAF2/6 interaction leading to IL-12 and IL-1B expression. Also linked to the inhibition of lysosomes fusion and GBP loading to the PVM	Truncated (and thus inactive) in some strains	Active	Active, but less than type II parasites	[174,198,199,205,206]
GRA16	TGGT1_208830	Nucleus	PP2A-B55, HAUSP	Modulates the expression of host cell genes involved in the control of cell-cycle progression, p53 signaling, steroids and lipids metabolism	Less active? (no virulence phenotype for the KO)	Active	Not studied	[189]
GRA18	TGGT1_288840	Cytoplasm	PP2A,GSK3,β- catenin	Activation of β-Catenin inducing upregulation of IFN-B1,CCL24 and anti-inflammatory chemokines CCL22 and CCL17	Active (secreted, but no functional study)	Active	Not studied	[191]
GRA24	TGGT1_230180	Nucleus	p38α/ MAPK	Activation of p38α/MAPK, inducing TH1/M1 polarization and cytokines/chemokines secretion	Active	Active	Not studied	[175]
GRA25	TGGT1_290700	PV	Unknown	Allow secretion of CXCL1 and CCL2 chemokines by infected macrophages	Not studied	Active	Less active	[176]
GRA28	TGGT1_231960	Nucleus	Unknown	Induces CCL22 secretion	Active	Likely active	Likely active	[177]
GRA60	TGGT1_204270	PVM	Unknown	Inhibits Irga6 and Irgb10 recruitment at the PVM	Active	Active	Not studied	[178]
HCE1/ TEEGR	TGGT1_239010	Nucleus	E2Fs/DP1	Inhibits NF-κB induced cytokines by interacting with host transcription factors and controls host Cyclin E expression by interacting with DP1	Active	Active	Not studied	[195,196]
TgIST	TGGT1_240060	Nucleus	NuRD, STAT1/2	Blocks signaling through type I interferon by recruiting the NuRD repressor and binding to STAT1/STAT2 heterodimers	Active	Active	Not studied	[193,194]
TgNSM	TGGT1_235140	Nucleus	NCoR	Inhibits interferon-regulated genes involved in cell death	Not studied	Active	Not studied	[190]
MAG1	TGGT1_270240	PVM/ Cytoplasm	Unknown	Inhibits IL-1β secretion in macrophages	Not studied	Active	Not studied	[179]
MAF1b	TGGT1_220950	PVM	MIB complex	Induces host mitochondria association with the PVM and modulates the cytokine response	Active	Inactive	Active	[86,180]

### ROP effectors

The first wave of effectors to be released are ROP proteins, which are discharged from the rhoptries into the cytoplasm of the host cell as invasion starts. Several ROPs are kinases that will be exported to the host cell nucleus in order to rapidly subvert signal transduction pathways governing the immune response or apoptosis. One example is ROP16, a tyrosine kinase that can phosphorylate host STAT3/STAT6 [181], which results in the inhibition of cytokine and NO production to favor parasite growth [182]. Another exported kinase, ROP38 is able to modulate the mitogen-activated protein kinase (MAPK) signaling pathway [183]. Several of the ROP effectors, once secreted into the host cytoplasm, associate with the cytoplasmic face of the PV to counteract effectors of host-cell autonomous immunity. The ROP5 pseudokinase, and its serine/threonine kinase partners ROP17/18 can for instance collaborate to phosphorylate IRGs and prevent their recruitment to the PV, again in a strain-dependent manner [184].

### GRA effectors

After the initial secretion of ROP factors, and as *T. gondii* tachyzoites establish themselves into the protective haven of their PV, they secrete GRA effectors. Most of those will remain within the confines of the PV (within the PV lumen or at the PVM) and will noticeably contribute to long-term nutrient acquisition [82,83]. Yet, some will be secreted into the host cell to act as modulators of important host pathways. Successful export of GRA effectors beyond the PVM usually depends on proteolytic processing by the ASP5 aspartyl protease, and then translocation through the PVM by a complex composed of MYR proteins [81,185,186]. Although translocation itself is not completely elucidated at the molecular level, it involves the aforementioned secreted and PVM-located ROP17 kinase [187], highlighting a collaborative effort between rhoptries and dense granules effectors to subvert host cell functions.

Six MYR-dependent secreted effectors have been described so far: GRA16, GRA18, GRA24, HCE1/TEEGR, TgNSM and TgIST [188]. These effectors will usually act in the host cell nucleus (with the exception of GRA18, that functions in the host cytoplasm), to modulate several pathways involved in parasite control. GRA16 impacts cell cycle-associated pathways through p53 modulation, and potentially promotes host cell survival under stress conditions [189]. Conversely, TgNSM inhibits IFN-regulated genes involved in host cell death, again likely promoting parasite growth and dissemination [190]. GRA18 induces a switch from a Th1 (cellular) to a Th2 (humoral) immune response, more likely to promote parasite survival [191]. GRA24 alters IL-12 levels and the IFNγ response through modulation of the p38 MAPK [192]. TgIST is also able to inhibit IFN-dependent signaling by acting as an inhibitor of the transcriptional activator STAT1 [193,194]. Finally, HCE1/TEEGR promotes parasite persistence by antagonizing the nuclear factor-kappa B (NF-κB) pathway [195,196], which plays an key role in modulating innate immunity, inflammation, but also cell death.

Some PVM-located GRAs also have important pro-survival functions for tachyzoites, like GRA7 that promotes IRG turnover [197]. Yet, the same protein, along with other PVM-located GRAs like GRA14 and GRA15, can also be an activator of the NF-κB pathway and promote macrophage activation in strains of *T. gondii* which are less virulent for mice [198,199].

### Selected examples of effector polymorphism and how strain-specificity influences T. gondii virulence

Strain-specific differences in *T. gondii* virulence can be linked to the fact that many effectors are highly polymorphic and thus have different outcomes depending on the strain (summarized on Table 1), but also of the host [17]. Type I *T. gondii* strains are much more virulent for mice than type II and III strains [14]. Taking advantage of these differences, powerful forward genetics approaches such as quantitative trait locus mapping have been used to identify strain-specific *T. gondii* virulence factors, including rhoptry kinases/pseudokinases ROP5, ROP16 and ROP18 [200]. As mentioned before, *T. gondii* interferes with the host immune response by a number of strategies ranging from direct local action on the host immune effectors, to modulation of upstream transcriptional programs in the host nucleus.

Direct interference with host anti-*T. gondii* factors is exemplified by ROP18 from type I strains, which is able to phosphorylate specific residues in the GTPase domain of IRGs to prevent their oligomerization and loading on the PVM, thus preventing its degradation [201,202]. Type III strains, on the contrary, do not express ROP18; thus, they are very sensitive to mouse IRGs [201,203]. Intriguingly, type II strains are efficiently eliminated by IRGs although they express ROP18, but it has been subsequently shown that ROP18 action is potentialized by the action of the ROP5 pseudokinase, which is less active in type II parasites [184]. If anything, this shows strain-specific virulence is the result of a complex and intricate interaction between several polymorphic effectors.

Two of the most striking examples of how strain-specific polymorphism modulates the inflammatory response in mice at the transcriptional level are ROP16 [181] and GRA15 [198]. In type I parasites ROP16 activates the SAT3/STAT6 pathway to favor parasite growth [182], while type II parasites contain a polymorphic form of ROP16 that is a poor STAT activator [204]. GRA15, which is more expressed in type II than in type III parasites, can stimulate the NF-κB-dependent production of inflammatory cytokines and activates macrophages [198,199], but is truncated and thus inactive in the type I lab-adapted RH strain. GRA15 also stimulates the recruitment of GBP1 to the PVM through an unknown mechanism [205]. However, it should be noted that the type I GT1 strain expresses a fully functional GRA15 [198,206]. This shows intra-clonal group variations can be observed and again, this highlights the complex and multi-factorial nature of host modulation by parasite effectors, whose differences include sequence polymorphism, but also changes in their expression levels.

The increasing number of effector proteins characterized over the recent years highlights the very complex nature of the host–pathogen interactions underlying *T. gondii* virulence in the mouse model. In any case, the lethal acute infection by type I parasites or chronic establishment of type II strains in mice constitute an archetypal and certainly useful model to study the balance of both pro- and anti-inflammatory modulation by various parasite proteins. The current state of knowledge shows there is a diversity of mechanisms governing this balance, allowing *T. gondii* to adapt to its host for disseminating without killing it. However, once again, how these effectors may co-opt host functions in other intermediate hosts, in particular in humans, certainly deserves to be investigated further. While murine immunity to *T. gondii* has been extensively studied, data available on the human immune response to the parasite shows common features, but also marked differences [207]. Because the hosts vary in some key innate immune pathways, parasite countermeasures are also likely adapted [208]. To refer to specific parasite effectors mentioned above, the function of the ROP5/17/18 complex on IRG recruitment to the PV is for example not conserved in the human host model [203], as humans lack the extensive IRG repertoire found in mice. While studies in the murine models have revealed a variety of parasite defense mechanisms and intricate relationship between *T. gondii* and its host, they need to be extended further to human cells. This may provide new insights into potential therapeutic strategies.

## Conclusions

*T. gondii* rarely cause serious disease in immunocompetent individuals, yet the high prevalence of this ubiquitous parasite makes it an important zoonotic pathogen. There is likely a coevolution between the parasite and its intermediate hosts, creating a balance between the acute and the chronic phases of the disease that favors parasite transmission. The domestic cat/mouse transmission cycle for *T. gondii*, that arose about 11,000 years ago as a consequence of human agricultural development and cat domestication, has likely been instrumental in selecting parasite strains infecting humans in the Old World [209,210]. However, humans themselves being rare or inaccessible prey for felids, they may be considered a dead-end for parasite transmission to the definitive hosts. While transmission between intermediate hosts by feeding, or vertical transmission to the progeny allow the parasite to bypass its sexual cycle in intermediate hosts felids cannot prey on, this limits genetic diversity and potentially reinforces the clonal population pattern. Perhaps as a result of all this, European infections are in large part attributed to strains that are few, homogenous, and mildly virulent. On the other hand, there is a greater diversity and pathogenicity of South American strains, which clearly contain more virulent variants. Human contamination with some of these strains originated from wild felids with a forest-based cycle, can cause significant damage and even death in adults who are not particularly immunocompromised, perhaps due to poor adaptation of the parasite to the host [15,211]. This is a sharp reminder that the balance between parasite virulence and adaptation for persistence in its hosts can be fragile.

First-line therapy against toxoplasmosis is usually a combination of pyrimethamine and sulfadiazine (drugs targeting folic acid metabolism), and it is used both for the treatment of congenital toxoplasmosis, or toxoplasmic encephalitis and ocular toxoplasmosis in adults [212]. Although relatively efficient in stopping tachyzoite proliferation, and thus acute toxoplasmosis, these treatments are inefficient against the encysted forms of the parasite [30]. More generally, drugs able to simultaneously target both developmental stages have not yet been identified. Also, there is currently no effective vaccine for human clinical use, which is another major tool unfortunately lacking in our arsenal to combat toxoplasmosis [213]. In any case, as we have seen in this review, *T. gondii* is master in the art of subverting the host immune system for ensuring its long-term persistence. Hence, although there is usually a robust cell-mediated immune response to primary infection in immunocompetent individuals, it controls but does not completely clear the parasite. Thus, so far no immunity sufficient for complete *T. gondii* elimination has been demonstrated in humans. While these are important challenges, there are prospects for developing new approaches for the control of human toxoplasmosis [214]. Recent progress on genetic manipulation of the parasites has allowed identifying novel factors conferring fitness and virulence to *T. gondii in vivo* [215–217]. Genetic tools, when coupled to computational modeling [218] or novel imaging techniques [219,220], can also reveal new potential drug targets. Besides, new *in vivo* or *in vitro* differentiation models [102,221–223] offer interesting perspectives for investigating different developmental stages that were largely understudied until now. All this will certainly allow an even broader understanding of the biology of *T. gondii*, which could lead to the identification of new anti-parasitic strategies.

## Data Availability

Data sharing is not applicable to this article as no new data were created or analyzed in this study.
